# Therapeutic effect of intranasal evaporative cooling in patients with migraine: a pilot study

**DOI:** 10.1186/1129-2377-16-5

**Published:** 2015-01-26

**Authors:** Jitka Vanderpol, Barbara Bishop, Manjit Matharu, Mark Glencorse

**Affiliations:** Neurology Department, Cumbria Partnership NHS Trust, Penrith, Cumbria UK; Institute of Neurology and The National Hospital for Neurology and Neurosurgery, Queen Square, London, UK; BeneChill International GmbH, Fritz-Vomfelde-Strasse 34, 40547 Düsseldorf, Germany

**Keywords:** Intranasal cooling, Migraine

## Abstract

**Background:**

Cryotherapy is the most common non-pharmacological pain-relieving method. The aim of this pilot study was to ascertain whether intranasal evaporative cooling may be an effective intervention in an acute migraine attack. Studies have previously demonstrated effectiveness of a variety of cryotherapy approaches. Intranasal evaporative cooling due to vascular anatomy, allows the transfer of venous blood from nasal and paranasal mucous membranes to the dura mater, thereby providing an excellent anatomical basis for the cooling processes.

**Methods:**

We conducted a prospective, open-label, observational, pilot study. Twenty-eight patients who satisfied the International Classification of Headache Disorders (ICHD 2) diagnostic criteria for migraine were recruited. A total of 20 treatments were administered in 15 patients. All patients provided pain severity scores and migraine-associated symptoms severity scores (based on a 0–10 visual analogue scale, [VAS]).

**Results:**

Out of the 20 treatments, intranasal evaporative cooling rendered patients’ pain and symptoms free immediately after treatment, in 8 of the treatments (40%), a further 10 treatments (50%) resulted in partial pain relief (headache reduced from severe or moderate to mild) and partial symptoms relief. At 2 hours, 9 treatments (45%) provided full pain and symptoms relief, with a further 9 treatments (45%) resulting in partial pain and symptoms relief. At 24 hours, 10 treatments (50%) resulted in patients reporting pain and symptom freedom and 3 (15%) provided partial pain relief. In summary 13 patients (87%) had benefit from the treatment within 2 hours that was sustained at 24 hours.

**Conclusions:**

Intranasal evaporative cooling gave considerable benefit to patients with migraine, improving headache severity and migraine-associated symptoms. A further randomised, placebo controlled, double blinded, parallel clinical trial is required to further investigate the potential of this application.

**Trial registration:**

Clinicaltrials.gov registered trial, ClinicalTrials.gov Identifier: NCT01898455.

## Background

Mechanical techniques to alleviate migraine symptoms have been used for many years, cooling and compression being the most frequently applied. Cryotherapy is the most common non-pharmacological self-administered pain-relieving method currently used by migraine sufferers [1]. The first manuscript documenting the application of ice mixtures was published by James Arnott in 1849 [2]. Simple techniques based on cryotherapy, using various methods of cold and ice application have been reported over the last three decades [3–6]. Friedman [7] described a device using hollow metal tubes cooled by circulating cold water, applied to the periapical area of the maxillary molars. Sprouse-Blum [8] reported benefit from applying frozen icepacks targeting carotid arteries at the front of the neck in migraine treatment.

Several pathophysiological mechanisms of action have been proposed. These mechanisms include:

1. *Neurovascular mechanism:* Application of cold induces vasoconstriction with subsequent decreased downstream blood flow [9, 10]. This leads to inhibition of the release of inflammatory mediators which results in decreased vascular permeability and local oedema [11, 12].
2. *Pain gating by differential effect on nerve conduction:* Cryotherapy induces analgesia by slowing nerve conduction velocity, with small myelinated fibers being affected first, followed by large myelinated fibers and the unmyelinated fibers being affected last [13, 14]. Via mechanism of the gate control of pain, affecting the small myelinated nociceptive pain afferents first, cryotherapy induces analgesia [15, 16].
3. *Metabolic mechanism:* Cryotherapy decreases metabolic and enzymatic activity, due to reduced demand for adenosine triphosphate (ATP) and oxygen [17].
4. *Transient Receptor Potential (TRP) channels:* Recent studies suggest that TRP channels may have a role in headache and pain genesis due to their response to environmental stimuli such as temperature changes [18].

The nasopharynx provides a large diffuse surface area that is highly vascular. Cooling via the nasopharynx therefore offers the ability to cool across the thin cribriform plate via both direct conductive mechanisms that do not rely on spontaneous circulation as well as indirect haematogenous mechanisms. Numerous subarachnoid and pial arterial branches exposed to the cerebrospinal fluid (CSF) have diameters in the range of the vessels of the retia mirabilia of animals in which selective brain cooling has been clearly established experimentally [19]. Vascular anatomy allows transfer of venous blood from the skin of the head as well as nasal and paranasal mucous membranes to the dura mater [20] thereby providing an excellent basis for the convective process of intranasal evaporative cooling. The dura mater, with its high vascularization, may transmit temperature changes to the CSF compartment, which may influence the temperature of brain parenchyma directly or indirectly, via brain arteries.

The aim of this study was to ascertain whether intranasal evaporative cooling might be an effective and safe intervention in acute migraine treatment.

## Methods

### Study design

This study is an investigator-initiated clinical trial, investigating the effectiveness of the RhinoChill^**©**^ intranasal cooling system for the acute relief of migraine in an adult population, through short periods of intranasal cooling.

We conducted a single-centre, prospective, open-label, observational, pilot study. Participants were enrolled from October 2013 to July 2014, from the outpatient clinics of the Neurology Department, Cumbria Partnership NHS Trust, and through a community engagement advertising campaign.

Study design consisted of a screening visit or phone call, baseline visit, minimum of one treatment and a follow-up phone call at 2 and 24 hours after each treatment. The principle of the intranasal cooling, procedure and safety aspects of the intervention were explained to each of the participants.

### Patient recruitment

The study participants were otherwise healthy adults who met ICHD 2 diagnostic criteria for episodic migraine with or without aura, or chronic migraine and were capable of giving informed consent. Exclusion criteria included history of any current co-morbid illness or difficulty with insertion of the nasal cannula due to congenital anatomical narrowing of the nasal passages.

### Trial device

**Figure 1:**
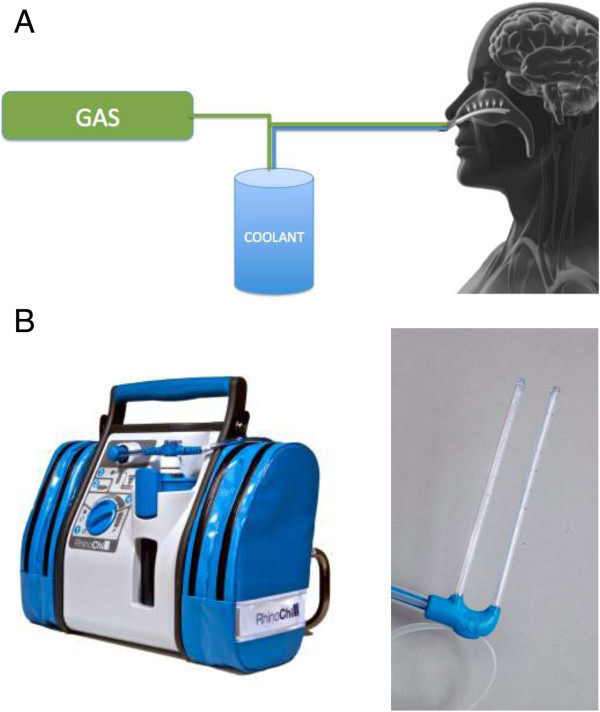
**RhinoChill©** **Intranasal cooling system: A/schematic, B/picture of the device and intranasal catheter.** Nasal catheter inserted into the patient’s nostrils, coolant driven by oxygen evaporates within the nasopharynx.

The RhinoChill^**©**^ Intranasal cooling device is a portable unit consisting of a control unit, a disposable nasal catheter and a 1-liter bottle of coolant. The device requires a supply of oxygen or medical air to drive the evaporation process of the coolant. The 10 cm long intranasal catheters have spray ports on the dorsal surface to distribute coolant into the nasal cavity, upwards towards the base of the skull (Figure 1).

The coolant used is perfluorohexane (PFH), a six-chain perfluorocarbon. PFH is a colorless, odourless, radiolucent liquid. PFH belongs to a class of perfluorocarbon fluids that are fully fluorinated with no functional reactive groups. They do not oxidize or hydrolyze and are thus chemically and biologically inert. They cannot undergo biological oxidation-reduction reactions to form reactive aldehydes, acid fluorides, radicals, or other acids. Since PFH does not react directly with biological tissue, they can form no reactive metabolites. PFH surface tension is lower than water so it will spread uniformly and quickly throughout the space in which it is sprayed. These properties facilitate RhinoChill coolant dispersion to the large diffuse surface area of the nasal cavity to maximize cooling.

Evaporation of the coolant provides rapid cooling to the nasal cavity of approximately 2°C, absorbing heat through evaporation and cooling local structures through direct conductive mechanisms and indirect haematogenous mechanisms. Safety of the device has been established in the cardiac arrest population where it has been used to induce rapid brain cooling for therapeutic hypothermia as neuroprotection [21, 22]. In this study, the device was only used on its lowest setting of ‘Low’ flow rate and for a maximum duration of 20 minutes as it was not the intention to induce therapeutic hypothermia.

### Therapeutic regimen

This trial was a feasibility study. To ensure patient safety, participants attended the hospital Day Case Unit during a migraine attack, to obtain their treatment under medical supervision. At onset of a migraine attack, the patient would contact the trial researcher and come into the hospital for treatment. On arrival, baseline observations were taken including infrared tympanic thermometry, non-invasive blood pressure (NIBP), pulse and oxygen saturation. Migraine pain severity was assessed using a visual analogue scale (VAS, 0–10 with 0 being pain free and 10 very severe pain), which was extrapolated to provide a categorical scale of pain intensity using the Glaxo-scale of Severe, Moderate, Mild and None. The use of both scales allow for comparison with other published literature regarding non-pharmaceutical and pharmaceutical approaches to acute migraine treatment. The associated migraine symptoms (nausea, vomiting, photophobia, visual symptoms, face asymmetry, neck pain/stiffness and numbness/weakness in extremities) were also scored using VAS, whereby the patient provided a subjective composite score regarding the overall severity of their combined associated symptoms.

Following confirmation of an acute migraine attack, treatment was commenced. Lubricated nasal cannulae were inserted into both nostrils. If full insertion proved intolerable then a shallow insertion only (as far as tolerable - ¼, ½ or ¾ of the cannula) was completed. A maximum of 20 minutes of cooling was provided with predetermined early stoppage if there was full relief of pain or at the participant’s request due to discomfort.

Migraine pain severity score, tympanic thermometry and NIBP, as well as pain and discomfort associated with the cooling device, was measured every 5 minutes throughout the treatment. Immediately after treatment was terminated, 2 hours post treatment and at 24 hours after treatment, pain severity and migraine-associated symptom composite scores were recorded. The participants were required to stay within the unit for a minimum of 1 hour following their treatment as a safety precaution. The patient was then phoned at 2 hours to enable recording of a migraine pain and symptom severity score.

The primary endpoint for this study was the reduction of pain and associated symptoms from baseline assessment, just after the treatment, at 2 and 24 hours post-treatment. Secondary endpoints were tolerance to RhinoChill cooling during treatment, pain and discomfort levels due to the cooling procedure itself, and adverse events noted throughout the treatment phase and during follow up.

### Statistical analyses

Data were analyzed using SPSS version 20.0 using paired sample *T*-test and the *χ*^2^ test, where p *<* 0.05 would be considered significant. Shapiro-Wilks and Friedman’s non-parametric test were used to test the validity of the pain and symptom severity VAS scores.

### Ethics or institutional review board approval

The study protocol was approved by the NRES Committee North West - Lancaster Ethics Committee. This study is registered at clinicaltrials.gov (NCT01898455). All participants provided written consent before taking part.

### Study protocol

Trial registration: Clinicaltrials.gov registered trial, ClinicalTrials.gov Identifier: NCT01898455.

## Results

Data are presented as an extended case series with qualitative data to support the acceptability aspects of the nasal cooling in migraine treatment. Data analysis was performed ‘per treatment’ due to the variation of treatment numbers received by the patients in the trial. However, overall treatment response per patient is also described.

### Demographic data

Twenty-eight adult patients were recruited into the trial of whom 15 patients received one or more treatments, providing 20 treatments in total. Eleven patients had a single treatment, while 3 patients had 2 treatments and 1 patient had 3. All patients were reviewed on entry to the clinic when they attended for treatment and asked about their current frequency of migraines. The most common reasons for lack of repeated treatments was due to migraines occurring outside of the operating hours of the trial, as well as inability to travel due to severity of migraine, not being able to leave work and having difficulty with childcare arrangements. The 13 patients who did not receive a treatment stated that they had migraines at times that were out-of-hours offered by the trial centre. The cohort comprised 12 (80%) female and 3 (20%) male patients, with mean age of 43 years (range 29–66 years). When asked about the normal duration of headache with standard medication and treatments, 14 (93%) patients stated that their headaches usually lasted longer than 24 hours after onset (Table 1).

**Table 1:** **Patient migraine characteristics**

Pt no.	Age	Sex	Time since migraine diagnosis	Migraine type	Duration of migraine attack	Severity of migraine VAS (0–10)
002	58	F	9 Years	Episodic Migraine without aura	24 – 48 hours	9
003	41	F	25 Years	Chronic Migraine	48 – 72 hours	10
004	66	M	29 Years	Chronic Migraine	24 – 36 hours	9
005	43	F	34 Years	Episodic Migraine without aura	24 – 72 hours	9
006	29	F	9 Years	Episodic Migraine without aura	12-72 hours	6
007	42	F	3.5 Years	Chronic Migraine	<12 hours	7.5
009	33	M	17 Years	Chronic Migraine	24 – 36 hours	3 – 9
010	57	F	4 Years	Episodic Migraine without aura	24 – 72 hours	7
012	36	F	2 Years	Chronic Migraine	48 – 72 hours	10
013	50	F	30 Years	Episodic Migraine with aura	48 – 72 hours	3 – 8
015	42	F	2 Years	Episodic Migraine with aura	36 – 48 hours	8
016	47	F	9 Years	Episodic Migraine with aura	24 – 48 hours	10
017	33	F	20 Years	Episodic Migraine without aura	24 – 36 hours	8 – 9
022	37	M	9 Years	Chronic Migraine	48 – 72 hours	7
023	30	F	4 Years	Chronic Migraine	48 – 72 hours	5 – 10

VAS, visual annalogue scale.

### Headache severity and migraine-associated symptom severity score

**Figure 2:**
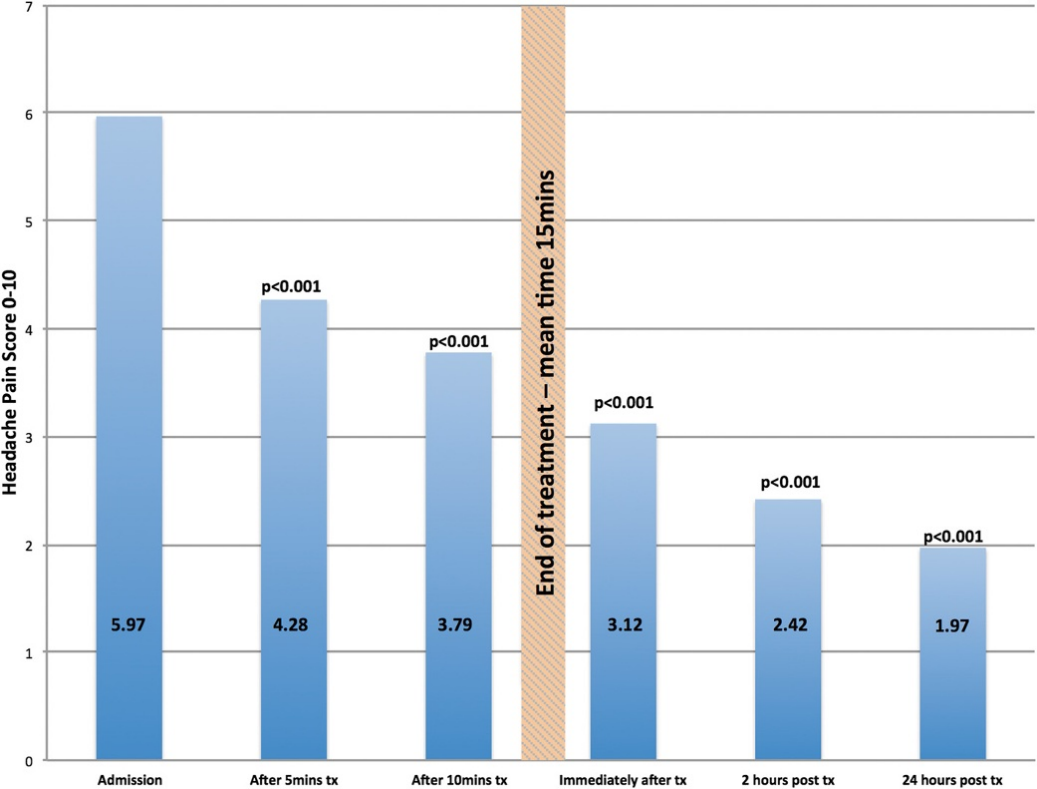
**Pain severity score (VAS 0-10).** p values correspond to comparison of pain severity score over time against baseline score. VAS: 0 = no pain/no discomfort, 10 = severe pain/discomfort. Tx = treatment.

**Figure 3:**
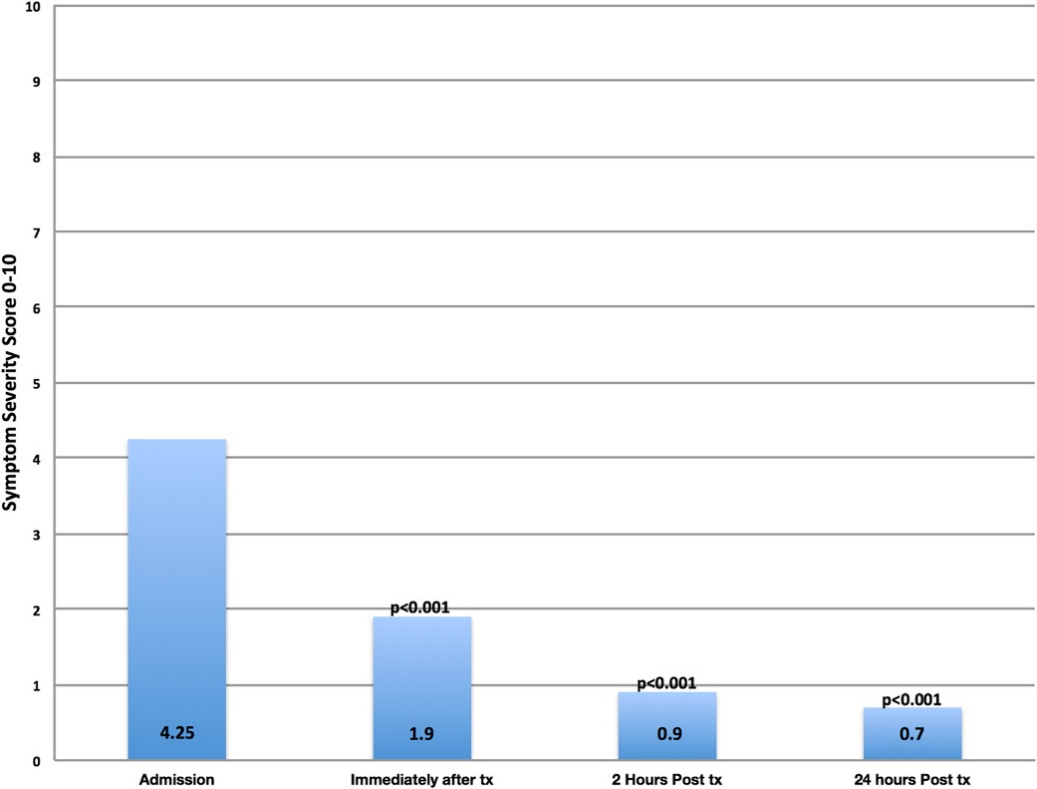
**Migraine-associated symptoms severity score (VAS 1–10).** p values correspond to comparison of symptom severity score over time against baseline score. VAS: 0 = no pain/no discomfort, 10 = severe pain/discomfort; Tx, Treatment.

All patients provided pain severity scores as well as a migraine-associated symptoms severity scores (single composite score) based on the visual analogue scale. This occurred on arrival to the department, throughout the treatment, immediately at the end of the treatment, at 2 and 24 hour post treatment. Results for pain scores and migraine-associated symptom severity scores are shown in Figures 2 and 3 and Tables 2 and 3.

**Table 2:** **Pain severity scores**

Time	Number	Pain severity measured using VAS (0–10)	
		Mean (±SD)	Range
On admission	20	5.98 (±1.84)	1-10
After 5 mins of treatment	20	4.28 (±2.62)	0-10
After 10 mins of treatment	20	3.79 (±2.81)	0-10
Immediately after treatment	20	3.13 (±2.73)	0-10
2 Hours post-treatment	20	2.43 (±2.28)	0-8
24 hours post-treatment	20	1.98 (±2.71)	0-9

VAS, visual analogue score; SD, standard deviation.

**Table 3:** **Migraine-associated symptoms severity scores**

Time	Number	Migraine-associated symptoms severity score measured using VAS (0–10)	
		Mean (±SD)	Range
On admission	20	4.25 (±2.37)	0-9
Immediately after treatment	20	1.90 (±1.83)	0-6
2 Hours post-treatment	20	0.90 (±1.24)	0-3.5
24 hours post-treatment	20	0.70 (±1.38)	0-5

VAS, visual analogue score; SD, standard deviation.

Out of the 20 treatments provided to patients in this trial, intranasal evaporative cooling rendered patients’ pain and symptoms free immediately after treatment in 8 of the treatments (40%). A further 10 treatments (50%) resulted in partial pain relief (headache reduced from severe or moderate to mild) and partial symptoms relief. At 2 hours, 9 treatments (45%) provided full pain and symptoms relief with a further 9 treatments (45%) resulting in partial pain and symptoms relief. At 24 hours, 10 treatments (50%) resulted in patients reporting pain and symptom freedom. Of those treatments remaining, 3 (15%) provided partial pain relief. Relapse of migraine after a period of relief occurred in 5 (25% of treatments). Two treatments (10%) did not respond to intranasal cooling.

When examining data on a ‘per patient’ basis rather than ‘per episode’, using mean scores for those patients who had one or more treatments and with the classification of treatment response as being a reduction in pain severity from severe/moderate to mild/none 13 patients (87%) had a positive treatment response to Intra-nasal cooling at two hours post-treatment that was sustained at 24 hours.

Comparing the group of patients suffering from chronic migraine (n = 7) with episodic migraine (n = 8), no difference was seen in treatment response rate albeit that the small numbers involved do not allow any firm conclusions to be drawn on any differences that there may be between treatment response in episodic and chronic migraine patients.

Reduction in both pain severity score and migraine-associated symptom severity score from baseline to each time point was statistically significant (p ≤ 0.001) representing a consistent downward trend in both pain and symptoms score.

Shapiro-Wilks test were carried out to test the validity of the pain and symptom severity VAS scores. For the pain and symptom severity score the distribution became less normal over time as the scores tended towards zero (as can be seen from the median values in Tables 2 and 3). To take this into account a Friedman’s non-parametric repeated measures test was used, where the overall statistics showed a significant effect. A series of Wilcoxon repeated measures test were calculated comparing scores on admission to scores immediately after treatment, 2 hours after treatment and 24 hours after treatment. Table 4 shows a significant overall effect for both Pain Severity Score and Symptom Severity Score, with all subsequent pairwise comparisons against the score on admission found to be significant.

**Table 4:** **Shapiro–Wilks and Friedman’s tests on pain scores and migraine-associated symptom scores**

	On admission (1)		Immediately after treatment (2)		2 Hours after treatment (3)		24 hours after treatment (4)			
	Mean (SD)	Median (SIR)	Mean (SD)	Median (SIR)	Mean (SD)	Median (SIR)	Mean (SD)	Median (SIR)	Friedman test (Chi-Square, df, p)	Wilcoxon pairwise comparisons
**Pain Score**	5.9(1.8)	6(5–7)	3.1(2.7)	3(0.6–5)	2.4(2.3)	2(0.3–3.5)	2.0(2.7)	0(0–4)	25.3(3),p < 0.001	1 < 2; 1 < 3; 1 < 4
**Symptom Score**	4.3(2.4)	5(3.2–5)	1.9(1.8)	2(0–3.4)	0.9(1.2)	0(0–2)	0.7(1.4)	0(0–1)	32.9(3),p < 0.001	1 < 2; 1 < 3; 1 < 4

SD, standard deviation; SIR, semi-Interquartile Range; df, degree of freedom.

### Tolerance of intranasal cooling

**Figure 4:**
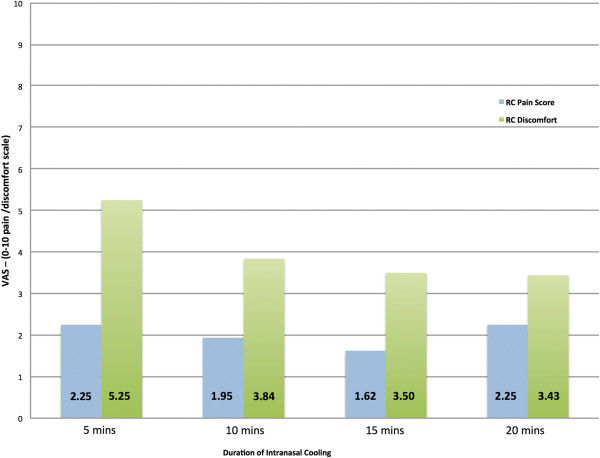
**Pain and discomfort associated with intra-nasal cooling.** VAS: 0 = No pain/Discomfort, 10 = severe pain/discomfort; RC, RhinoChill.

Patients provided visual analogue scores during the application of Intranasal cooling to describe any pain and/or discomfort associated with the cooling process itself, independent of migraine pain and associated symptoms scores. Mean scores are presented in Figure 4 and Table 5. Shallower insertions were associated with less pain and discomfort. Patients also rated benefits of the intranasal cooling compared to discomfort or pain associated with the treatment. Two patients (13%) reported that the discomfort/pain outweighed any benefit delivered. Thirteen patients (87%) would use the treatment if available outside of the trial and 2 (13%) would not. Furthermore, when asked how Intranasal cooling compared to current rescue medication taken, 9 patients (60%) stated that intranasal cooling was better, 4 (26%) stated the response was as good as their current medication and 2 (14%) felt that their current rescue therapies worked better than the intranasal cooling.

**Table 5:** **Pain and discomfort associated with intranasal cooling on the Visual Analogue Scale (VAS)**

Variable	N	Min	Max	Mean	Std. deviation
RC Associated pain at 5 mins	20	.00	8.00	2.25	2.95
RC Associated pain at 10 mins	19	.00	8.00	1.94	2.80
RC Associated pain at 15 mins	13	.00	7.00	1.61	2.66
RC Associated pain at 20 mins	8	.00	7.00	2.25	3.15
RC Associated discomfort at 5 mins	20	.00	10.00	5.25	2.65
RC Associated discomfort at 10 mins	19	.00	8.00	3.84	2.29
RC Associated discomfort at 15 mins	13	.00	8.00	3.50	2.27
RC Associated discomfort at 20 mins	8	.00	8.00	3.44	2.58

RC, RhinoChill; Min, Minimum value recorded; Max, Maximum value recorded; Std. Deviation, Standard Deviation; VAS: 0 = no pain/no discomfort, 10 = severe pain/discomfort.

### Tympanic temperature

Due to the use of a short duration low flow treatment with the RhinoChill device, we did not note decrease in tympanic temperature after a maximum of 20 minutes of intranasal cooling on a low flow setting (36.65°C baseline v 36.60°C post treatment. p = 0.485).

### Mean arterial blood pressure

**Figure 5:**
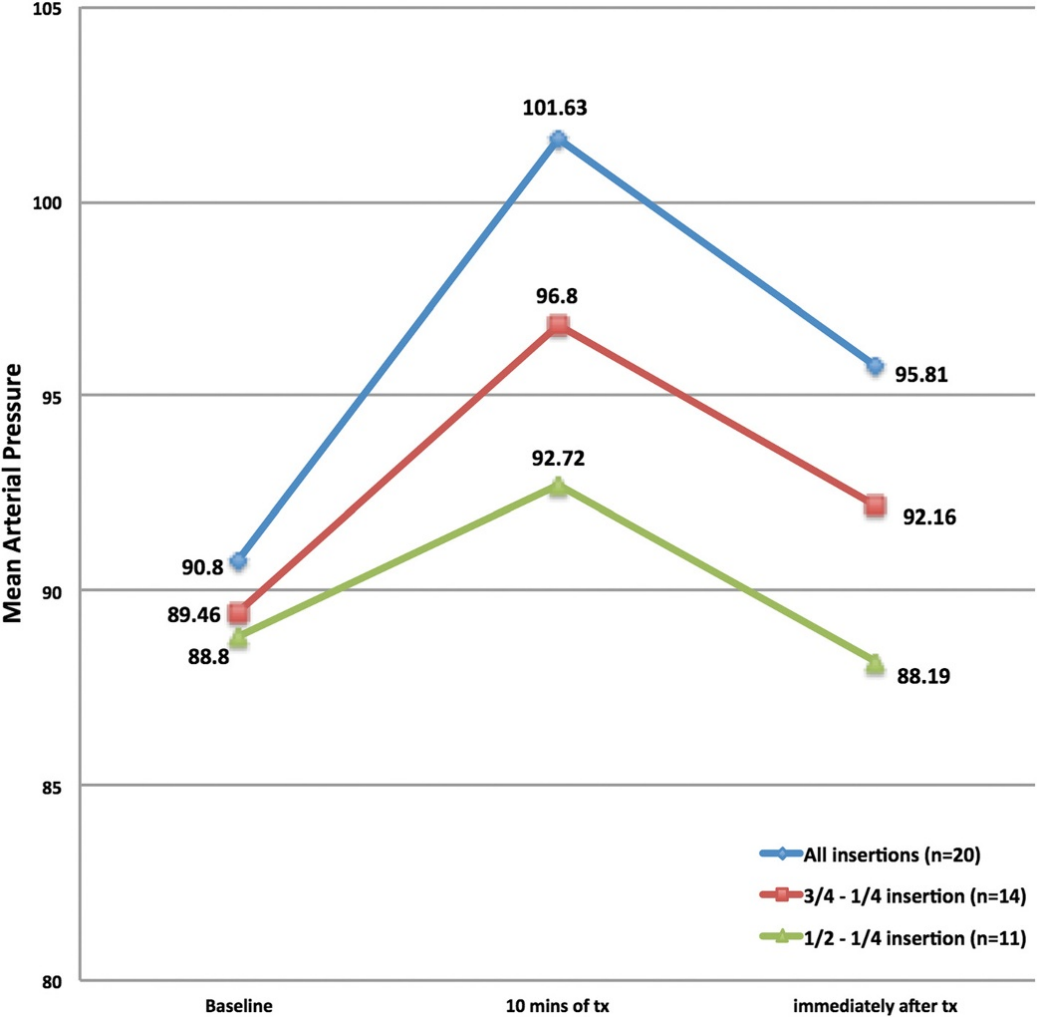
**Mean arterial pressure and depth of insertion of nasal catheter.** Tx, Treatment; MAP, Mean Arterial Pressure.

When looking at mean arterial pressure across all treated migraine episodes (n = 20), the difference between baseline mean arterial pressure (MAP) and 10 minutes into treatment as well as baseline MAP and immediately after treatment are both significantly different (Baseline v 10 mins, p = <0.001; Baseline v immediately after treatment, p = 0.04). As catheter insertion depth is decreased, the difference between MAP readings also starts to decrease (Figure 5). MAP for patients who had an insertion depth of ¼ to ¾ of the nasal catheter (n = 14) remained significant when comparing baseline to 10 minutes of treatment (p = 0.02) but became non-significant when comparing to immediately after treatment (p = 0.32). Finally, when examining data from those patients who had only a ¼ or ½ insertion (n = 11), there is no difference between baseline and 10 minutes of treatment (p = 0.147) or immediately following treatment (p = 0.78).

### Adverse events

Adverse events recorded are listed in Table 6. During the treatment nasal discomfort and pain due to catheter was reported in 3 treatments, nasal discomfort due to cooling was reported in 2 treatments, mild epistaxis (blood streaks reported in 1 case), and excess coolant dripping from nose was reported during 2 treatments. Hypertension was noted in one patient on two consecutive treatments within 10 minutes of starting intranasal cooling with a full depth insertion of the nasal catheter (increase of NIBP from 146/88 to 192/111 – treatment 1; and from 141/82 to 184/102 – treatment 2). At the time of measurement, this patient scored a high pain and discomfort score associated with the catheter placement. Treatment was discontinued early in one case. Blood pressure immediately decreased following treatment and returned to baseline within 1 hour following treatment. Following this patient’s experience, further treatments were offered with a shallower insertion, either ¾, ½, or ¼ depth depending on patient comfort. No further episodes of hypertension were recorded once shallower insertions were delivered.

**Table 6:** **Adverse events**

Adverse event during treatment	Number of episodes
Nasal Discomfort/Pain due to catheter	3
Nasal discomfort due to cooling	2
Hypertension	2
Mild epistaxis	1
Discomfort due to excess fluid dripping from nose	2
Adverse Event Following Treatment	
Transient recurrent sneezing	2
Runny nose	2
Strange taste	1
Strange smell	1
Dry eyes	1
Dizziness	1
Sinus pressure	1

Early termination due to side effects was required in 5 treatments. This included discomfort due to excess non-evaporated coolant dripping out of the nose (two), discomfort/pain related to nasal catheter placement (two), in one case together with transient hypertension, and one episode of mild epistaxis (blood streaked nasal mucous). In four of these episodes, despite early termination, the patients still recieved a treatment benefit. Out of those patients who terminated early, 3 out of the 5 stated that they would use the device again if it was available within the NHS.

All side effects were mild and subsided spontaneously, without need for further treatment. No treatment related side effects were reported at the 24 hour follow up call.

## Discussion

This pilot trial demonstrated a significant therapeutic effect with intranasal evaporative cooling as an acute treatment for migraine. The study findings suggest that intranasal cooling rapidly relieves pain as well as migraine-associated symptoms.

Migraine is considered to be a neurovascular disorder. Even though the event initiating the actual migraine attack remain unknown, activation of the trigeminovascular system is considered to play an essential role in pathophysiology of the migraine attack [23]. We believe that the vascular anatomy of the nasal cavity allows transfer of venous blood from paranasal mucous membranes to the dura mater and may transmit temperature changes via both direct conductive mechanisms that do not rely on spontaneous circulation as well as indirect haematogenous mechanisms via dural vessels. This may influence the temperature of the brain parenchyma directly or indirectly, via brain arteries, possibly inducing vasoconstriction, reducing blood flow and inhibiting release of inflamatory mediators. These inflammatory mediators play a role in increased vascular permeability and local oedema [11, 12].

The effect of cooling might have impact on metabolic and enzymatic activity, albeit it is not clear what the mode of action is which impacts on the release of local neuropeptides.

Recent studies indicate that Transient Receptor Potential (TRP) channels may have a role in the headache and pain genesis due to their response to stimuli such as temperature, changes in osmolality, pH and environmental products [18, 24, 25]. TRP channels may represent novel targets for headache therapeutics. Out of the six subfamilies of TRP channels, Transient Receptor Potential V1, M8, A1 (TRPV1, TRPM8, TRPA1) seems to be the ones researchers believe are expressed on the external nociceptors, which could result in activation of nociceptors and produce pain [26–28].

Intranasal cooling might have had a stimulating effect on TRP channels, likely in antagonistic inhibitory way for TRPV1, TRPA1, and possibly as an agonist on TRPM8 channel.

Other inhibitory factors such as differential alteration of nerve conduction velocities mediated its effect via gate control mechanism, might have played a part in the process leading to the pain and symptoms termination.

A vasoconstrictor response of brain vessels to 100% oxygen inhalation has been known for decades. In patients with acute hemispheric infarction, regional vasoconstrictor responsiveness to 100% oxygen inhalation was decreased [29]. Whilst it is clear that oxygen is a very useful therapy in cluster headaches, there is some evidence of the benefit of hyperbaric oxygen therapy in relieving acute migraine, but the benefit of normobaric oxygen therapy in migraine remains rather controversial [30].

The coolant, a non-ozone depleting six chain perflurocarbon, perfluorohexane (PFH), does not oxidize or hydrolyze and cannot undergo biological oxidation-reduction reactions to form reactive aldehydes, acid fluorides, radicals, or other acids. Since it does not react directly with biological tissue, and cannot form reactive metabolites or react with receptors, or channels, so we anticipate PFH doesn’t have any direct benefit in the migraine treatment, other than acting as a coolant.

The therapeutic effect of intranasal evaporative cooling was not diminished by the shallow insertion of catheter, nor impacted by premature termination of the treatment. With respect to the duration of the treatment and depth of the nasal catheter insertion, further evaluation is required involving a study with a larger number of participants.

Our trial highlighted the importance of a non-pharmacological approach. Further trials to examine a non-pharmacological approach are warranted.

We acknowledge that a major limitation of this study is the small sample size and lack of a control group. A further randomized, placebo controlled trial is therefore essential to confirm the effectiveness of intranasal cooling as an acute treatment in migraine.

## Conclusion

In conclusion we found that there is a therapeutic effect from intranasal evaporative cooling in patients with migraine, giving benefit on headache as well as accompanying migraine symptoms to 13 patients (87%) at 2 hours post-treatment; even though this was an in-hospital treatment with a device and thus the placebo effect may be pronounced.

This study did not reveal any significant or severe side effects. Nasal cooling was well tolerated by majority of the participants and we found that nasal cooling was safe to use for this indication.

While these results are promising, a randomized placebo controlled, double blinded trial is needed to confirm the efficacy of intranasal cooling as an acute treatment in migraine. Further studies will also be required to clarify the mode of action of this therapeutic intervention.

## Abbreviations

CSF: Cerebrospinal fluid
NIBP: Noninvasive blood pressure
VAS: Visual analogue scale
MAP: Mean arterial pressure
ICHD 2: International Classification of Headache Disorders
TRP: Transient Receptor Potential channels
PFH: Perfluorocarbon – perfluorohexane
CGRP: Calcitonin Gene-related peptide.
